# Monthly Prices of Substances Used in Pharmacy

**Published:** 1814-05

**Authors:** 


### *Monthly Prices of* SUBSTANCES *used in* PHARMACY,

At Messrs. SELWAY and HENLEY's (Chemical and Pharmaceutical Laboratory,) 35, Upper Mary-le-bone Street, Portland Place.

*(To be continued regularly.)*

| | s. | d. |
|---|---|---|
| Acetum Scillæ ........... lb. | 2 | 0 |
| Abietis Resina ........... lb. | 2 | 3 |
| Acaciæ Gummi ............ | 2 | 3 |
| ——— elect. ...... | 3 | 3 |
| Acidum Aceticum .... cong. | 3 | 8 |
| ——— Benzoicum ...... lb. | 6 | 0 |
| ——— Oxalicum .......... | 18 | 0 |
| ——— Boracicum ........ | 24 | 0 |
| ——— Nitricum .......... | 4 | 6 |
| ——— Nitrosum .......... | 2 | 6 |
| ——— Muriaticum ........ | 0 | 8 |
| ——— Sulphuricum ...... | 0 | 6 |
| ——— Arom. .. | 5 | 0 |
| Alcohol sp. gr. 815 M. lb. | 5 | 6 |
| Æther sulphur. sp. gr. 789 | 10 | 0 |
| ——— rectificatus sp. gr. 744 | 12 | 0 |
| Ærugo .................. lb. | 7 | 0 |
| Aloes spicatæ extractum .... | 5 | 6 |
| ——— vulgaris extractum .... | 4 | 4 |
| Alumen .................. | 0 | 4 |
| ——— exsiccatum ........ | 0 | 8 |
| Ammoniæ Murias .......... | 2 | 4 |
| ——— Carbonas ........ | 3 | 6 |
| ——— Nitras .......... | 5 | 6 |
| Amygdalæ Amaræ ........ | | |
| ——— Dulces .......... | 5 | 8 |
| Ammoniacum (Gutt.) ...... | 6 | 6 |
| ——— (Commun.) .. | 3 | 6 |
| Anthemidis Flores (com.) .. | 1 | 9 |
| ——— (opt.) .... | 2 | 0 |
| Antimonium .............. | 3 | 0 |
| Antimonii Sulphuretum .... | 1 | 0 |
| ——— Oxydum ........ | | |
| ——— Murias ........ | 3 | 9 |
| Antimonium Tartarizatum .. | 5 | 0 |
| Arsenici Oxydum .......... | 0 | 10 |
| Argenti Nitras ............ | 6 | 9 |
| Assafœtida Gummi-resina .. | 6 | 9 |
| Aurantii Cortex .......... | 2 | 6 |
| Balsamum Peruvianum ...... | 33 | 0 |
| ——— Tolutanum ...... | 15 | 0 |
| Barytæ Murias ............ | 6 | 0 |
| Benzoinum elect. .......... | 11 | 0 |
| Bismuthum .............. | 3 | 0 |
| ——— Oxydum ...... | 16 | 0 |
| Calamina præp. .......... | 0 | 3 |
| Calumbæ Radix .......... | 2 | 6 |
| Calx .................... | 1 | 0 |
| Cambogia opt. ............ | 9 | 6 |
| Camphora ................ | 9 | 6 |
| Canella Cortex .......... | 3 | 4 |
| Cardamomi Semina opt. .... | 12 | 0 |
| Cascarillæ Cortex ........ | 6 | 6 |
| Castoreum ............ unc. | 4 | 4 |

| | s. | d. |
|---|---|---|
| Catechu Extractum ........ | 2 | 3 |
| Cetaceum ................ | 3 | 0 |
| Cera flava .............. | 3 | 6 |
| ——— alba .............. | 3 | 0 |
| Ceratum Cetacei .......... | 3 | 0 |
| ——— Resinæ .......... | 2 | 8 |
| ——— Calamina ........ | 3 | 0 |
| ——— Plumbi superacet. .. | 4 | 0 |
| ——— Lyttæ .......... | 4 | 0 |
| ——— Sabinæ .......... | 2 | 8 |
| ——— Plumbi Carb. .... | 3 | 0 |
| ——— Comp. .... | 3 | 8 |
| ——— Saponis .......... | 5 | 0 |
| Cinchonæ cordifoliæ Cortex (yellow) ...... | 9 | 0 |
| ——— lancifoliæ Cortex (quilled) ...... | 13 | 0 |
| ——— oblongifoliæ Cortex (red) ........ | 16 | 0 |
| Cinnamomi Cortex ........ | 16 | 0 |
| Coccus (Coccinella) ........ | 64 | 0 |
| Colocynthidis Pulpæ .... M. | 8 | 0 |
| Copaiba .................. | 7 | 0 |
| Colchici Radix .......... P. | 3 | 7 |
| Croci stigmata ........ unc. | 4 | 0 |
| Cupri sulphas .......... lb. | 1 | 0 |
| Cuprum ammoniatum ...... | 6 | 0 |
| Curcuma ................ | 2 | 6 |
| Cuspariæ Cortex .......... | 4 | 0 |
| Confectio Aromatica ........ | 8 | 6 |
| ——— Aurantii ........ | 2 | 9 |
| ——— Opii ............ | 4 | 6 |
| ——— Rosæ Caninæ .... | 1 | 8 |
| ——— Gallicæ .... | 2 | 0 |
| ——— Scammoniæ ...... | 15 | 0 |
| ——— Sennæ .......... | 2 | 0 |
| Emplastrum Ammoniaci .... | 8 | 0 |
| ——— Cumini ........ | 2 | 0 |
| ——— Galbani Comp. .. | 2 | 6 |
| ——— Lyttæ .......... | 6 | 6 |
| ——— Opii .......... | 2 | 6 |
| ——— Plumbi ........ | 1 | 6 |
| ——— Resinæ ........ | 1 | 6 |
| ——— Roborans ...... | 2 | 0 |
| ——— Hydrargyri .... | 2 | 6 |
| Extractum Anthemidis .. unc. | 1 | 0 |
| ——— Belladonnæ ...... | 1 | 0 |
| ——— Cinchonæ .. unc. | 3 | 0 |
| ——— resinos. | 4 | 0 |
| ——— Colocynthidis .... | 2 | 0 |
| ——— comp. | 1 | 0 |
| ——— Conii ............ | 0 | 6 |
| ——— Gentianæ ........ | 0 | 5 |
| ——— Glycyrrhizæ .. lb. | 3 | 0 |

Extractum

| | s. | d. |
|---|---|---|
| Extractum Hæmatoxyli ·· unc. | 0 | 5 |
| —— Humuli | 0 | 6 |
| —— Hyoscyami | 1 | 0 |
| —— Jalapæ 1*s*. 6*d*. Res: | 2 | 8 |
| —— Opii | 3 | 6 |
| —— Papaveris | 0 | 6 |
| —— Rhæi | 2 | 4 |
| —— Sarsaparillæ | 0 | 10 |
| —— Taraxaci | 0 | 6 |
| Ferri carbonas lb. | 0 | 9 |
| —— sulphas | 1 | 0 |
| Ferrum Ammoniatum | 3 | 6 |
| —— Tartarizatum | 4 | 0 |
| Galbani Gummi-resinæ | 7 | 6 |
| Gentianæ Radix | 1 | 0 |
| Guaiaci Resina | 6 | 0 |
| Hydrargyrus purificatus | 5 | 6 |
| —— cum Creta | 4 | 0 |
| Hydrargyri Oxymurias | 8 | 0 |
| —— Submurias | 8 | 6 |
| —— Hydros. | 10 | 0 |
| —— Nitrico-Oxydum | 8 | 6 |
| —— Oxydum Cinereum | 16 | 0 |
| —— Rubrum | 5 | 0 |
| —— Sulphuretum Nig. | 3 | 6 |
| —— Rub. | 8 | 0 |
| —— Præcipitatus Alb. | 8 | 0 |
| Hellebori nigri Radix | 2 | 6 |
| Ipecacuanhæ Radix | 22 | 0 |
| —— Pulvis | 23 | 0 |
| Jalapæ Radix | 6 | 0 |
| —— Pulvis | 6 | 6 |
| Kino | 12 | 0 |
| Liquor Plumbi Acetatis M. lb. | 1 | 0 |
| —— Ammoniæ P.L. | 2 | 8 |
| —— Arsenicalis | 2 | 0 |
| —— Potassæ | 0 | 9 |
| —— Vol. Corn. Cerv. | 1 | 3 |
| Linimentum Camphoræ comp. | 5 | 6 |
| —— Saponis comp. | 5 | 6 |
| Lichen Islandicus P. lb. | 1 | 9 |
| Lyttæ | 13 | 6 |
| Magnesia | 8 | 6 |
| —— Carbonas | 3 | 0 |
| —— Sulphas | 1 | 3 |
| Manna optima | 8 | 6 |
| —— communis | | |
| Mel Rosæ | 2 | 6 |
| —— Despumat | 3 | 0 |
| Moschus pod, 24*s*. in gr. unc. | 40 | 0 |
| Mastiche lb. | 5 | 6 |
| Myristicæ Nuclei | 25 | 0 |
| Myrrha elect. | 6 | 6 |
| Olibanum | 3 | 0 |
| Opoponax | 23 | 0 |
| Opium (Turkey) | 34 | 0 |
| —— (East India) | | |
| Oleum Amygdale lb. | 5 | 4 |
| —— Anisi unc. | 2 | 6 |
| —— Anthemidis | 5 | 0 |

| | s. | d. |
|---|---|---|
| Oleum Cinnamomi Cassiæ | 8 | 0 |
| —— Caryophili | 5 | 0 |
| —— Cajuputi | 8 | 0 |
| —— Carui | 1 | 3 |
| —— Juniperi | 0 | 9 |
| —— Lavandulæ | 4 | 0 |
| —— Lini cong. | 8 | 6 |
| —— Menthæ piperitæ unc. | 4 | 6 |
| —— viridis | 3 | 0 |
| —— Olivæ cong. | 35 | 0 |
| —— secundum | | |
| —— Origani unc. | 2 | 0 |
| —— Pimentæ | 4 | 6 |
| —— Ricini optim. (per bottle) | 10 | 0 |
| —— Rosmarini unc. | 0 | 9 |
| —— Succini 2*s*. 4*d*. rect. | 2 | 6 |
| —— Sulphuratum M. lb. | 1 | 6 |
| —— Terebinthinæ | 2 | 0 |
| —— rectificatum | 2 | 0 |
| Oxymel Scillæ | 3 | 0 |
| Papaveris Capsulæ (per 100) | 2 | 4 |
| Plumbi Carbonas lb. | 0 | 8 |
| —— Superacetas | 2 | 2 |
| —— Oxydum semi-vitreum | 0 | 6 |
| Potassa Fusa unc. | 0 | 6 |
| —— cum Calce | 1 | 6 |
| Potassæ Nitras lb. | 1 | 0 |
| —— Acetas | 8 | 0 |
| —— Subcarbonas | 1 | 2 |
| —— Carbonas | 4 | 0 |
| —— Sulphas | 1 | 0 |
| —— Sulphuretum | 1 | 6 |
| —— Supersulphas | 3 | 0 |
| —— Tartras | 3 | 0 |
| —— Supertartras | 2 | 0 |
| Pilulæ Hydrargyri | 6 | 0 |
| —— Aloes comp. | 8 | 0 |
| —— cum Colocynth. | 16 | 0 |
| —— Saponis cum Opio | 18 | 6 |
| —— Scillæ Compos. | 6 | 6 |
| —— e Styrace | 48 | 0 |
| Pulvis Antimonialis | 6 | 6 |
| Quassiæ Ras | 2 | 4 |
| Resina Flava | 0 | 5 |
| —— Nigra | 0 | 8 |
| Rhæi Radix (Russia) | 30 | 0 |
| —— (East India) | 12 | 0 |
| Rosæ Folia | 10 | 6 |
| Sapo (Spanish) | 2 | 6 |
| Sarsaparilla Radix | 5 | 0 |
| Scammoniæ Gum-Resina unc. | 2 | 3 |
| Scilla Radix siccata lb. | 4 | 6 |
| Senegæ Radix | | |
| Sennæ Folia | 6 | 0 |
| Serpentariæ Radix | | |
| Simaroubæ Cortex | 4 | 0 |
| Sodæ Boras | 3 | 9 |
| —— Carbonas | 7 | 0 |
| —— Subcarbonas | 1 | 4 |
| —— exsiccata | 2 | 8 |

| | s. | d. |
|---|---|---|
| Sodæ Sulphas | 0 | 8 |
| ——— Phosphas | 2 | 8 |
| ——— Acetas ... unc. | 0 | 10 |
| Soda tartarizata ... lb. | 2 | 4 |
| Spongia usta | 20 | 0 |
| Spiritus Ammoniæ ... M. lb. | 4 | 0 |
| ——— aromaticus | 4 | 6 |
| ——— fœtidus | 3 | 6 |
| ——— succinatus | 4 | 6 |
| ——— Cinnamomi | 3 | 6 |
| ——— Juniperi C. | 3 | 0 |
| ——— Lavandulæ | 4 | 6 |
| ——— Myristicæ | 3 | 0 |
| ——— Pimentæ | 3 | 0 |
| ——— Rosmarini | 3 | 6 |
| ——— Ætheris Aromaticus | 6 | 0 |
| ——— Compositus | 6 | 0 |
| ——— Nitrici sp. gr. 860 | 5 | 6 |
| ——— Sulphurici | 6 | 0 |
| ——— Vini rectificatus | 28 | 0 |
| Syrupus Aurantii | 1 | 8 |
| ——— Rutæ | 1 | 8 |
| ——— Papaveris | 2 | 0 |
| ——— Violæ | 2 | 10 |
| ——— Rhei | 2 | 10 |
| ——— Rhamni | 2 | 0 |
| ——— Croci | 3 | 0 |
| Sulphur Sublimatum | 0 | 6 |
| ——— Lotum | 1 | 0 |
| ——— Præcipitatum | 1 | 0 |
| Tamarindus opt. | 2 | 6 |
| Terebinthina Vulgaris | 0 | 9 |
| ——— Canadensis | | |
| ——— Chia | | |
| Tragacantha Gummi ... lb. | 10 | 0 |
| ——— Pulvis | 10 | 6 |
| Tinctura Aurantii ... M. lb. | 4 | 0 |
| ——— Aloes | 4 | 0 |
| ——— Composita | 6 | 0 |
| ——— Assafœtidæ | 5 | 6 |
| ——— Balsa. Tolut. | 5 | 6 |
| ——— Benzoini Comp. | 5 | 6 |
| ——— Camphoræ Comp. | 4 | 0 |
| ——— Cardamomi | 4 | 0 |
| ——— Comp. | 4 | 6 |
| ——— Calumbæ | 4 | 0 |
| ——— Capsici | 4 | 0 |
| ——— Cascarillæ | 4 | 6 |
| ——— Castorei | 7 | 0 |
| ——— Catechu | 4 | 0 |
| ——— Cinchonæ | 6 | 0 |
| ——— Compos. | 6 | 0 |

| | s. | d. |
|---|---|---|
| Tinctura Cinchonæ Ammon. | 7 | 0 |
| ——— Croci | 6 | 6 |
| ——— Digitalis | 4 | 6 |
| ——— Ferri Ammoniati | 4 | 0 |
| ——— Cinnamomi | 4 | 6 |
| ——— Comp. | 4 | 6 |
| ——— Gentianæ Comp. | 4 | 0 |
| ——— Guaiaci | 6 | 0 |
| ——— Ammon. | 6 | 0 |
| ——— Hellebori Nigri | 4 | 6 |
| ——— Humuli | 5 | 0 |
| ——— Hyoscyami Nig. | 4 | 6 |
| ——— Jalapæ | 4 | 6 |
| ——— Japonica | 4 | 6 |
| ——— Kino | 4 | 6 |
| ——— Lyttæ | 4 | 0 |
| ——— Myrrhæ | 4 | 6 |
| ——— Opii | 6 | 6 |
| ——— Camphorat. | 4 | 6 |
| ——— Quassiæ | 4 | 0 |
| ——— Rhei | 4 | 6 |
| ——— Comp. | 4 | 0 |
| ——— Scillæ | 4 | 6 |
| ——— Sennæ | 4 | 6 |
| ——— Serpentariæ | 6 | 0 |
| ——— Valerianæ | 4 | 6 |
| ——— Amm. | 5 | 6 |
| ——— Zingiberis | 4 | 6 |
| Valerianæ Radix | 1 | 8 |
| Veratri Radix | 1 | 6 |
| Unguentum Hydrargyri-fortius | 5 | 0 |
| ——— Nitratis | 3 | 3 |
| ——— Nitrico-oxy. | 2 | 9 |
| ——— Sulphuris Comp. | 2 | 0 |
| ——— Sambuci | 2 | 0 |
| ——— Virid | 1 | 8 |
| ——— Altheæ | 2 | 0 |
| ——— Cetacei | 3 | 6 |
| ——— Veratri | 1 | 8 |
| ——— Zinci | 2 | 0 |
| ——— Hyd. præcip. Alb. | 3 | 0 |
| ——— Picis | 1 | 6 |
| Uvæ Ursi Folia | 2 | 4 |
| Vinum Aloes | 5 | 6 |
| ——— Antimoniale | 3 | 0 |
| ——— Ferri | 3 | 6 |
| ——— Ipecacuanhæ | 5 | 6 |
| ——— Opii | 8 | 0 |
| Zinci Oxydum | 4 | 6 |
| ——— Sulphas purif. | 2 | 0 |
| ——— Acetas ... unc. | 1 | 6 |
| Zincum ... lb. | 1 | 6 |
| Zingiberis Radix opt. | 2 | 4 |

English Leeches 14s. per dozen.

Foreign Leeches, in general as good as the English, 36s. to 40s. per hundred.

*Price of Vials per Gross.*—8 oz. 70s.—6 oz. 58s—4 oz. 47s.—3 oz. 43s.—2 oz. 36s.—1 oz. 30s.—$\frac{1}{2}$ oz. 24s.

METEO-

